# Conceptual Framework of the Impact of Health Technology on Healthcare System

**DOI:** 10.3389/fphar.2019.00933

**Published:** 2019-09-03

**Authors:** Samar F. Farid

**Affiliations:** Clinical Pharmacy Department, Faculty of Pharmacy, Cairo University, Cairo, Egypt

**Keywords:** healthcare system, eHealth, health technologies, mHealth, electronic health records, telemedicine, e-prescribing, technology-enabled pharmacy

## Abstract

The World Health Organization (WHO) promotes health systems strengthening as a means of improving population health, especially in low- and middle-income countries. The United Nations Sustainable Development Goals highlight the importance of investing in workforce development to improve population health and economic well-being. In relation to pharmaceuticals, health systems face challenges in terms of i) guaranteeing access to needed drugs, ii) rationalizing medicines use, and iii) avoiding harm from adverse events. There is a pressing need to better understand the relationships between technology and pharmacy practice when strengthening pharmaceutical care systems. In response, this paper examines ways in which harnessing new technologies can change pharmacy practice and strengthen pharmaceutical systems for the benefit of patients. The paper will present a conceptual framework as well as exploring case studies.

## Introduction

Every human has the right to the healthcare they need, to interventions that enable them to live healthy (Porter, 2010). Provision of healthcare differs substantially between countries especially with the presence of different healthcare systems and insurance. More developed economies with larger resources usually have higher levels of health and well-being (Cameron et al., 2009; Godman et al., 2017). Worldwide, the healthcare environment is changing. It is becoming increasingly obvious that affordable high-quality healthcare cannot be delivered without harnessing new ways of delivering care (Kvedar et al., 2014; Godman et al., 2018). Using new technologies is a promising solution to help cope with current challenges and to improve healthcare and pharmacy practice (Kvedar et al., 2014).

“Technology” may be defined as the “dynamic clustering of techniques, methods, skills and processes used in the production of goods or services or in the achievement of outcomes that deliver desired benefits for consumers” (Baines et al., 2018). New technologies are having an impact on healthcare delivery. Health technology is defined by the WHO as “the application of organized knowledge and skills in the form of devices, medicines, vaccines, procedures and systems developed to solve a health problem, improve quality of lives” (World Health Assembly, 2007).

For instance, the internet is allowing new healthcare solutions to emerge that improve communication and information sharing by patients and healthcare providers. Web-based electronic communications are proliferating in many ways including all sectors of civilization, for instance, e-mail, e-commerce, e-prescribing, e-health, etc. (Mackert et al., 2014; Srivastava et al., 2015). Other new technologies are emerging. Electronic devices, telecommunications, wireless, and audio-visual systems are being constantly developed as information and communication technology (ICT) in health (Henriquez-Camacho et al., 2014). The application of internet, web technology, and ICT in health is termed e-health (Van De Belt et al., 2010; Mackert et al., 2014). Areas of e-health include electronic health records (EHR), e-prescribing, telemedicine, telehealth, consumer health informatics, and m-health (Henriquez-Camacho et al., 2014).

E-health, telemedicine, telehealth, m-health, and digital health are all interchangeable terms included under the umbrella of “technology-enabled care” (TEC) (Ryu, 2012b). For instance, new technologies designed to support pharmacists have been called “technology-enabled pharmacy” (Baines et al., 2018). Technology-enabled care involves using health technology, smartphones’ applications, voice and written messages, Bluetooth technology, digital media, automated robots, remote monitoring devices, and wearable technology for effective integration of care (Ryu, 2012a). Delivering care *via* those new technologies changes the ways by which information and health services are delivered which leads to patient-focused healthcare and personalization. The following are examples of how new technologies improve the healthcare delivery (Kvedar et al., 2014):

- Getting access to specialty physicians, specialized knowledge, and time-place independent services enables us to overcome geographic boundaries.
- Remote diagnosis *via* interactive videoconferencing and remote consultation *via* counseling software or applications could overcome the space barrier and waiting time spent in clinics to meet physicians.
- Remote Intensive Care *via* Tele-ICU technologies can positively affect intensivist coverage over ICUs leading to decreased mortality and ICU lengths-of-stay.
- Medication adherence *via* smartphone reminder applications, internet-connected alarms for pill caps, or printed calendars on medication blister improves patients’ compliance leading to fewer clinical problems and less costly care over time.
- Reducing Referral Wait Times *via* e-referral service model could leverage specialist capacity.

However, no matter how promising that looks for patients’ drug adherence and healthcare, we have to consider patients’ adaptation to these ways of technologies stressing on uneducated or poorly educated populations; otherwise it all goes in vain (Nashilongo et al., 2017; Rampamba et al., 2018).

The need for harnessing new technologies in healthcare and pharmacy practice is increasing. Their impact in enhancing patient–provider’s satisfaction, improving quality of life, participating patients’ on their own care and health directly, reducing cost, ensuring efficacy, enhancing the professional scope of pharmacy practice and extending care to wider population is significant (Kvedar et al., 2014; Baines et al., 2018). Adopting new technologies could lead to a renaissance of pharmacy practice taking drug dispensing to a new level of appropriateness and overcoming the massive consequences of its misuse (Baines, 2015; Markovic-Pekovic et al., 2017). Shedding light on the ways by which new technologies can impact healthcare and benefit the patient will allow health systems to strengthen their quality, releasing new innovations and paving the way to overcome any existing or potential challenges.

E-Health market becomes a huge business that offers a great potential in delivering care. The slow-paced field of healthcare is in need to join the global digital flow of technology. We can see the great impact of fast-paced digital technology on every detail of our life. Harmony of this fast-developing industry with the healthcare industry can dramatically change the healthcare delivering market (The Rise of mHealth Apps: A Market Snapshot—Liquid State).

The main issue that motivates this research article is poor availability of affordable and effective healthcare technologies in the developing countries. Although various technologies are available or under investigations, we still need technologies or to develop new ways to make them more accessible, affordable, effective, easy to use, and even capable of addressing problems of drug persistence and adherence in case of noncommunicable diseases (NCDs) (Howitt et al., 2012; Masum et al., 2013).

Analysis of each project and developed e-health experiment in developed and developing world is a must to overcome challenges and try innovating new ways transforming healthcare. Therefore, this article overviews the impact of healthcare technologies on healthcare system in developed and developing world, discussing the pros and cons trying to find our pace in harnessing new ways for better healthcare delivery. Focusing on the situation in developing countries and in Egypt has a very crucial role in improving the structure of healthcare market in that world.

## Health Technologies in Developed and Developing Countries

### Electronic Health Record

EHR is defined as a collective electronic record of the patients’ health information that includes the patient medical history, medication prescriptions, physical examinations, medical reports, and notes of the healthcare professionals ensuring the standardized readable complete orders (Campanella et al., 2016). EHR as a growing healthcare innovation is associated with many benefits and challenges (Chao et al., 2013). One of the major benefits of EHR is the storage of patients’ medical information in addition to the savings that could occur in the paper resources and the space needed for the documentation (Chao et al., 2013; Kalogriopoulos et al., 2009).

EHR could also allow patients’ medical information sharing leading to more efficient workflow among the departments of the health institutes (Shachak et al., 2009; O’Malley et al., 2010; Chao et al., 2013).

Another advantage that EHR could offer is the speeding up of the diagnosis and clinical decision process through making all the diagnostic results accessible once available. All of these advantages can be added to the medication repetition and drug allergies that could be avoided by the convenient proper access to patient medical records (Chao et al., 2013). On the other hand, EHR might carry some challenges for the patients, physicians, and health institutions (Chao et al., 2013). For patients, the main concern about EHR lies in the privacy issues in addition to the risk of less eye contact and face-to-face communication due to the physician putting information into the system during consultation (Zhang et al., 2012; Chao et al., 2013).

Regarding the physicians, they might be faced with some challenges on using EHR as it might be a time-consuming system (Abramson et al., 2012; Chao et al., 2013; Pollard et al., 2013). Also the risk of the health data loss, illegal information leakage, and the high cost associated with adopting the system are the three main drawbacks for the health institutes as stakeholders (Kalogriopoulos et al., 2009; Chao et al., 2013; Syzdykova et al., 2017).

Because of the many advantages stated previously, many of the developed countries such as United States, Canada, and United Kingdom put a target of paperless hospitals *via* a stepwise transition from paper-based health records to EHR in their healthcare settings (Al-Aswad et al., 2013). Some developing countries have also adopted EHR systems as a trial to improve their healthcare services, such as Kenya and Peru (Kalogriopoulos et al., 2009). In 2001, an EHR system was developed in Kenya that serves about 60,000 patients. The beneficial impact of this system was the shortening in the length of patient visits, reduced provider time per patient, and less waiting time spent by the patients in the clinic (Fraser et al., 2005).

Another example in Kenya is Kenyatta National Hospital. In this hospital, all patients’ data are entered electronically by the medical record staff into the health information system that could help in the data retrieval of any data in the future (Kivoto et al., 2018).

### Electronic Prescribing

E-prescribing is defined as an electronic computer-based transmission and filling of a prescription instead of paper prescriptions that assist healthcare providers to electronically submit or renew a prescription authorization to the community pharmacy that includes a two-way transmission between the care provider and the dispenser (eHealth Initiative Foundation, 2008; Electronic prescription drug program., 2012). There are many pros and cons for the e-prescribing. Both the patient and the prescriber can benefit from such intervention (eHealth Initiative Foundation, 2008; Klepser et al., 2016). For the patient, e-prescribing can improve the patient safety and quality of care through a number of ways such as elimination of illegible handwriting for paper-based prescriptions, avoiding oral miscommunications, presence of safety checkers, and accessibility to the patient medical and medication history. All of these can eventually reduce medication errors and the resultant adverse drug events (Varkey et al., 2007; eHealth Initiative Foundation, 2008; Abramson et al., 2012).

For the prescribers, e-prescribing allows reducing the time spent on call backs and faxing for clarifications in addition to improving the prescriber mobility and convenience as it can be used through mobile devices (eHealth Initiative Foundation 2008; Taylor et al., 2008; Klepser et al., 2016). For the medical governmental institutions, e-prescribing offers a great benefit as in case of drug recall as it allows finding all the patients with particular prescription (eHealth Initiative Foundation, 2008). All of these benefits can be added to the valuable input of the e-prescribing in improving patients’ compliance through eliminating the effort of dropping off a paper prescription and reducing the obstacles in the prescription filling process in addition to providing the appropriate alternatives with lower cost that indeed can improve patient adherence to medications (eHealth Initiative Foundation, 2008; Arlington, 2012).

In sum, the implementation of e-prescribing can overcome many paper-based prescription problems leading to cost savings, increased illegibility, reduced medication errors, the reduced need for redundant paperwork, and better access to the different drug information and patient medication history, eventually leading to improving therapy outcome (Varkey et al., 2007; Taylor et al., 2008; Yu et al., 2009; Devine et al., 2010).

Despite of all the previously listed pros, there are challenges that have hindered the more widespread adoption of e-prescribing as high financial cost and the longer time it may take for the return of investment for small practices especially in rural areas (eHealth Initiative Foundation, 2008; Lander et al., 2013; Porterfield et al., 2014). Since there is no absolute confidence of the completeness and the accuracy of patient medication history available in online records, this puts a burden on the prescriber to validate such information and to update the record accordingly (eHealth Initiative Foundation, 2008). Another patient-related challenge regarding e-prescriptions is patient acceptance issues as some patients do not feel comfortable with e-prescriptions (Greenberg et al., 2004; eHealth Initiative Foundation, 2008).

In an American study, Kaushal et al. assess the effectiveness of an e-prescribing system on medication errors with respect to prescribing errors (Institute of Medicine (US) Committee on Quality of Health Care in America, 2000; Kaushal et al., 2010). This study showed the potential benefit of the e-prescribing system in improving patient safety through the (statistically significant) reduction in the prescribing errors.

Also in Sweden, many measures have been introduced within the last few years to enhance the quality and efficiency of prescribing. One of these measures was the implementation of e-prescribing that was agreed as one of the long-term strategies to ensure rational, safe, and cost-effective treatments (Godman et al., 2009).

On assessing the acceptance of e-prescribing adoption in an Asian developing country (Pakistan), it was found that the main concern about its adoption is the associated high financial costs accompanying its implementation (Kaushal et al., 2010).

### Telehealth and Telemedicine

Telehealth or telecommunication enables the bi-directional transfer of information among the different partners of the healthcare system over considerable distances using text, voice, video, or images (DA and Allen, 1995; Ryu, 2012a; Goundrey-Smith, 2014). Telemedicine provides high-quality care despite socioeconomic and geographical barriers, and facilitates home monitoring and treatment (Hjelm, 2005; Kahn, 2015). The patient can send and receive any modifications in his/her care plan through many mobile applications or even through remote nursing supplied by electronic devices and laptop (Bellazzi et al., 2001; Vontetsianos et al., 2005). Through telemedicine, the healthcare provider can remind the patients to take the medications at the proper time improving medication adherence (Ansari and Fong, 2006). Confidentiality and security issues are the most common challenges facing system developers. They have to ensure that transmitted information won’t be modified or accessed by unauthorized persons (Hjelm, 2005; Ansari and Fong, 2006). Most available systems evaluating the impact of telemedicine focus on patient and physician perceptions and acceptance rather than short-term clinical outcomes (Kahn, 2015). Another challenge facing physicians is that they can’t perform an entire physical examination through video call (Hjelm, 2005). Telemedicine is integrated in healthcare systems since the early 1990s by both developed and developing countries through many ways. For example, integration of wireless oscillometric home blood pressure monitoring with smartphone technology in the United States demonstrated a greater improvement in blood pressure control (Ciemins et al., 2018). In addition, BP readings are not uploaded by the patient but uploaded automatically at the time of measurement eliminating the probability that patients might alter or select the BP readings.

In Bangladesh, they assessed the application of a Portable Health Clinic. This system involves three phases: first the personnel has to register to get an ID; second, get examined by physician and the checkup data automatically sent and saved in a Portable Health Clinic server. Finally, the computer analyzes all the data and categorizes the individual health conditions into four categories (healthy, caution, affected, and emergency). The patient in need was connected audio-visually by a physician located in the clinic (Nakashima et al., 2013).

Another example is the application of Telemedicine in the Eastern Province of Saudi Arabia, the e-Health Center established in the King Faisal Specialist Hospital & Research Centre (KFSH&RC) in Riyadh. The e-Health Center was implemented in order to facilitate access to medical consultations and to spread healthcare educational activities. The percentage of adopting telemedicine in Saudi Arabia is low. This was attributed to the lack of knowledge about the concept of telemedicine and its application and benefits. The most frequently cited challenges besides the lack of knowledge are the difficulty in applying telemedicine due to financial concern and weak infrastructure and the lack of time to adopt telemedicine (El-Mahalli et al., 2012).

### Automated Dispensing

An area of development in healthcare system is the application of automated dispensing in hospitals in order to improve the medication use process and to reduce medication errors. Automated dispensing involves the use of automated dispensing cabinets (ACDs). ACDs are computer-controlled devices providing secure storage units; access to the medication is restricted to holders of electronically readable keys or by fingerprint (Pharmaceutical dispensing cabinet., 1979). Automated dispensing has been shown to reduce the incidence of dispensing errors, improve the efficiency and speed of the dispensing process, provide a more secure storage for narcotics, reduce the space occupied by drug storage, and reduce workload on pharmacists and nurses (Fitzpatrick et al., 2005; Chapuis et al., 2010). However, nurse attitude toward automated medication dispensing may affect its effectiveness despite the implementation of a well-designed system (Novek et al., 2000). Also, ACDs still account for 15% of dispensing errors, although ACD requires pharmacist review of drug orders before drug access; the devices have an “override system” option in case of emergency, which can be misused by inadequately trained persons. *Alphabetic drug pick list* feature may contribute to dispensing errors, with nurses confusing between two drugs with similar spelling, or similar appearance, if present in the same drawer. Also, concentration errors occur between adult and pediatric doses for the same medications stored in AC (Paparella, 2006; Gaunt et al., 2007).

The following devices are examples of automated dispensing.

The Pyxis Medstation, an automated device operating like bank-teller machines placed in nursing units and linked to the hospital computers. All drug orders and patient file transferred on the device are readable by authorized persons. This system is designed to record all transactions and charges that are automatically loaded on patient bills (Lee et al., 1992; Borel and Rascati, 1995).

The McLaughlin dispensing system, a bedside locked dispensing device, loaded with medications for every patient and programmed to unlock automatically at an appropriate time of administration (Barker et al., 1984).

United Arab Emirates has launched robot auto-dispensing pharmacy at Al Fujairah Hospital in early 2017, Nadd Al Hamar Health Center, two smart pharmacies at Rashid Hospital in early 2018, another in Latifa Hospital, and one in Dubai Hospital. The robot at Al Fujairah Hospital can dispense up to 2,000 of pharmaceutical packs/hour and can store up to 45,000 medications at one time. Also, it can follow the expiry date of medications and give a clear image about monthly and annual needs and consumptions. The robot has saved patients’ waiting time and also saved more time for pharmacists for consultations and instructions rather than dispensing (Dubai’s “Smart Pharmacy” robot auto-dispenses medicine in two minutes | News | Time Out Dubai, 2019; UAE Ministry of Health & Prevention Issues a Report on Robotic Pharmacy Project Achievements, 2019).

Our search didn’t retrieve any information about the impact of automated dispensing in LMICs, which may be due to the high cost needed to implement this kind of technology.

### Barcode Medication Administration

Barcode medication administration consists of handheld device scanning patient identification wristbands and medication barcodes. This is connected to medication records allowing providers to verify appropriateness of medication before administration. If the scanned medication doesn’t correspond to the physician order, the device generates a warning sound or light (Koppel et al., 2008; Morriss et al., 2009). Barcode medication administration ensures the five rights of medication administration: right patient, drug, dose, route, and time (Morriss et al., 2009). Implementation of medication barcode scanning helps in reducing dispensing errors caused by ADCs (Gaunt et al., 2007) without introducing new types of error (Seibert et al., 2014). As the case with ADCs, the effectiveness of the technology depends on personnel use; also some technical issues, for example, non-readable torn or missed barcodes, malfunctioning scanners, or failing batteries hinder its appropriate use and delay drug administration (Yang et al., 2012).

Integration of barcode administration system to the auto-dispensing pharmacy at United Arab Emirates’ hospitals had achieved zero dispensing errors in 2017 and 2018 (Dubai’s “Smart Pharmacy” robot auto-dispenses medicine in two minutes | News | Time Out Dubai, 2019).

Unfortunately, similar to automated dispensing, no information was found about the impact of barcode medication application on healthcare system in LMICs.

## Healthcare Service Status in Egypt

Several organizations and ministries share the organization and decision-making of healthcare systems in Egypt. These organizations include the Ministry of Health and population (MoHP), Health Insurance Organization (HIO), Curative Care Organization, and Educational Hospitals Organization (Farid, 2017). Hospitals in Egypt are classified into public and private. The public hospitals are classified into three types: university hospitals, health insurance hospitals, and MoHP hospitals (Eldin et al., 2013). Healthcare in Egypt faces many problems including the quality of healthcare services, the increase in out-of-pocket expenditure for those services (which has reached 55% of total spending), growing population, and the lack of updated infrastructure and trained personnel (Farid, 2017). Only half of the Egyptians have health insurance. In 2014, the per capita expenditure on health sector reached $178 annually. The government is required to spend at least 3% of gross domestic product (GDP) on healthcare (UPR Briefing, 2014). A new health insurance law is drafted to ensure a more comprehensive system of health services (Farid, 2017).

## Health Technology in Egypt

Egypt has made a progress in providing an ICT infrastructure and legal framework (Hussein and Khalifa, 2012). The WHO eHealth profile of Egypt published in 2011 described the progress of eHealth applications in Egypt (World Health Organization (WHO), 2010). The main eHealth foundation actions taken in Egypt include developing supportive eHealth policies and providing sufficient funding and developing the ICT infrastructure. The Egyptian government together with the MoHP and the Ministry of Communication and Information Technology (MCIT) worked on eHealth programs to provide better health services to the Egyptians. These programs include (Hussein and Khalifa, 2012; Khedr and Alsheref, 2014):

- **National Network for Citizen Health:** It aimed to develop central treatment management, direct patients to different therapeutic units, develop information system and databases of the central department for citizen health treatment, and connect all peripheral departments by a virtual private network.
- **Emergency Medical Call Center and Ambulance Center:** The MoHP and MCIT cooperated to establish the Emergency Medical Service (EMS) in Greater Cairo and provide the ambulance service with a computerized ambulance dispatch system.
- **Information System Units in public hospitals:** It aimed to establish information system units in 700 hospitals nationwide. The units are used to facilitate patients’ registration, financial and administration operations, and training medical staff.
- **National Healthcare Capacity Building Project:** This project aimed to create a pool of competent healthcare professionals by providing training to MoHP staff. The training program included: Basic Information Technology (IT) skills, Biomedical Informatics Professional Training, and biomedical awareness. The project would also include a central unit that links hospitals and keeps medical records.
- **National Cancer Registry Program:** It utilizes state-of-the-art data mining technologies to find health indicators for investigating reasons of the cancer spreading. The city of Aswan was selected initially to be enlisted in the program.
- **National Picture Archiving and Communications System (PACS) Project:** Launched in 2010 and aimed to develop a centralized database PACS and Radiology Information System (RIS) that integrates clinical images and scanned documents into the patient’s EMRs. A local database was developed in Fum El Khalij (the main Center of Excellence) and the eight hospitals included in the project.

Other programs include the following: Pilot Project for Hospital Automation, IT Health Master Plan, Integrated Health Record System, and Regional Center for Women’s Health in Alexandria (Hussein and Khalifa, 2012).

## Other eHealth Applications in Egypt

### Electronic Health Records

Over the last years, the government tried to implement EHR nationwide. However, some of these trials failed for several reasons. These trials were as follows (Abdelgaber et al., 2017):

**a- First trial:** An agreement was made between Data Management System Company and HIO to apply EHR and hospital administration systems. It was implemented initially in Suez hospital, but was stopped in 2010 for financial and political reasons.
**b- Second trial:** An agreement was made between Siemens Company and HIO to conduct EHR. It was implemented initially in Abu El-Rish children hospital. The system had some technical and administrative barriers and was stopped in 2010 for financial reasons.
**c- Third trial:** In this trial the Egyptian HealthCare Accreditation Program was made to make all hospitals accredited over time. Also medical records conduction was a condition for accreditation. However, it was not a condition to implement it electronically. But, it is still a good standardized start. Currently, four hospitals were accredited by MoHP and many others are working on it.

According to Khedr and Alsheref, the following public hospitals contain EHR systems: Ain Shams University Hospital, Kasr Al Ainy French Hospital, National Cancer Institute, and Nasser Institute Hospital; as well as the following private hospitals: Al Salam Al Dawly Hospital, Al Salam Hospital, and El Ganzouri Hospital. Those systems contained patient demographics, financial reports, appointments, lab data, and pharmacy data as databases. Those systems had the following limitations: lack of standardization, no system for nursing, physician examination, nor radiology; the lab systems and patient databases were not connected (Khedr and Alsheref, 2014).

## Limitations for Applying EHR in Egypt and Future Recommendations

A questionnaire-based study on 50 hospitals showed that only around 7% adopted EHR system (Eldin et al., 2013). The main barriers for system adoption were costs (81%), lack of technical support (44%), and lack of standards (44%). In hospitals that adopted EHR, the main impacts were increasing quality of service (100%), improving performance (67%), and increasing physicians’ time efficiency (50%).

A Strengths, Weaknesses, Opportunities, and Threats (SWOT) analysis made by Abdelgaber et al. showed the main barriers for EHR implementation in Egypt. Those included high cost, lack of clear time plan, lack of IT infrastructure, technical ability and training, lack of standardization, privacy concerns, and resistance for new technology (Abdelgaber et al., 2017).

Implementation of EHR in Egypt will improve quality of healthcare, decrease medication errors, facilitate the coordination between healthcare professionals and settings, and provide a fast access to patient information (Thakkar and Davis, 2006). In order to apply an EHR nationwide network, we need to establish specialized EHR Management Board and EHR training authority, find a source of funding, set up the security criteria and accreditation procedures, and announce the benefits of EHR to the society (Abdelgaber et al., 2017).

### Telemedicine

According to the MCIT, by the end of February 2018, there were 100.24 million mobile subscribers (total Egyptian population is 99,414,614) while the mobile penetration rate was 110.19%, and 37.9 million internet users while the internet penetration rate was 44.3% (Ministry of Communications and Information Technology – Egypt, 2018). Egypt has progressed in committing to intellectual property rights agreements, creating the National Telecommunications Regulatory Authority (NTRA) and issuing communications laws for liberalizing the communications sector in Egypt. The Egyptian Information Society worked on providing various applications for government, education, business, and healthcare (Hussein and Khalifa, 2012).

In the past years, some telemedicine projects have been implemented. Those include:

- **Arab-African Telemedicine Network Initiative:** Launched in 2002 by International Telecommunication Union (ITU). It aimed to form a multi-country telemedicine network between participating countries to prevent and treat diseases. The expected participating countries included Egypt, Jordan, Libya, Morocco, Ethiopia, Tunisia, Sudan, Mali, and Uganda. The first phase aimed to connect two medical centers in US and Europe (Hussein and Khalifa, 2012).
- **Inter-hospital tele-consulting project between Palermo and Cairo:** Launched in 2002 by ARNAS-Civic Hospital of Palermo and Italian Hospital Umberto I in Cairo. It aimed to establish a network for tele-consulting and exchanging data of complicated cases between the two hospitals (Hussein and Khalifa, 2012).
- **Egyptian Telemedicine Network (ETN):** Launched by MoHP and MCIT in 2006. The first phase consisted of seven telemedicine units. Those units provided many healthcare services as radiology and ECG. In 2007, two mobile units were used for screening breast cancer. However, after 1 year, the project faced many financial, legal, and technological problems (Sultan, 2006).
- **Women HealthCare Mobile Unit Project:** Launched in 2007 by the MoHP and MCIT to screen ladies over 45 years by mobile and fixed digital mammography imaging units for free. The RIS is used for registering the patients’ demographics. Then, the mammograms are sent to the national radiology center of excellence. The radiologists there use PACS and RIS for diagnosis and reporting to the units (Eldin et al., 2013). By the end of 2010, the project consisted of 10 mobile units and 11 fixed units. Each unit was planned to receive 50–80 cases per day. Till today, more than 60,000 cases were checked.
- **TeleMedic @ Egypt Project:** Launched in 2009, by the Information Technology Institute (ITI)–MCIT. It was funded by the Holding Company for Biological Products and Vaccines—VACSERA (Egypt), the Research, Development and Innovation Program (RDI), and the Institute for Biomedical Engineering IBMT (Germany). It aimed to provide tele-consultation services for infectious diseases in unequipped areas. The project is conducted in the Family Care Unit of Mahsma in Ismailia and provides preventive, diagnostic, and public health services (Hussein and Khalifa, 2011).
- **Pan Africa Project:** Launched in 2009 by the MCIT, Alexandria University, Telecommunications Consultants India Limited and the regional center for health development. Video-conference sessions were held between 12 hospitals in India and the healthcare organizations in Alexandria to provide tele-consultations. Also, the project provided eLearning programs (Hussein and Khalifa, 2012).
- **Saving children through tele-consultation in remote Egypt:** This was a 1-year pilot (2009–2010) conducted by MCIT, the private sector in Egypt, the technical capacity of the Child and Adolescent Health Unit (CAH) of WHO-EMRO, and UNDP development knowledge of the country. It connected the Pediatric Department of the El Shatby Hospital–Alexandria and Siwa main hospital. It aimed to provide tele-consultation services and eLearning of physicians. Forty-five children were helped by this project (El Tokali et al., 2009).

There have been other trials for launching telemedicine applications by different parties. For example:

- In 2008, a paper by Elgharably et al. presented the prototype of a system that allows physicians to monitor and diagnose patients especially the elderly over the Internet. A patient could measure his vital signs through a module connected wirelessly *via* Bluetooth to his computer and then send his data through the internet or through an SMS to be saved on a database in the healthcare organization. At any time the doctor could access the data of his patient, chat with him, or consult with another doctor. The system needs further work to improve its functionality and to be able to manufacture. This will necessitate making it integrable with HIS, PACS, and RIS (Elgharably et al., 2008).
- In 2016, mDiabetes program was launched as a part of a larger mHealth program for chronic non-communicable diseases. It was run by a partnership between the MoHP, MCIT, and Ministry of Higher Education and Scientific Research (MHESR), WHO, and the ITU, known as Be He@lthy Be Mobile. This initiative aimed to use mobile technology to minimize illness and reduce the social and economic burden due to non-communicable diseases. Participants in the program would get regular SMS on lifestyle choices and steps about how to manage their diabetes. The first phase was planned to send 10,000 SMS. A second phase, later in 2016, was planned to cover a larger number of diabetics. It was planned in a later phase to send SMS on methods for diabetes prevention for the general population. Also, it was planned to provide a two-way interactive SMS service where recipients can select the information they need (WHO.EMRO, 2016).
- In 2017, Heshmat et al. proposed a prototype mobile application called (YourClinic). It can be used through smartphones by patients, physicians, and nurses in outpatient clinics. Patients can use it to search for their preferred clinics and then select the preferred day and time. Also the patient can easily submit his medical complaints and an online consultation will be done for him. Physicians can use the application to know their future schedule for the next week. The nurse then coordinates with the physician and the patients. The expected impacts of the application upon patients, doctors, and nurses are decreasing waiting time, patients stress, and healthcare staff working hours. As a future work, a business model for this application will be developed (Heshmat et al., 2017).

Currently, there is another mobile application used as a platform for reserving consultation appointments in outpatient private clinics called Vezeeta for Doctors. It was downloaded by more than 100,000 users (Google play store, 2018).

## Limitations for Applying Telemedicine in Egypt and Future Recommendations

Although Egypt has done many telemedicine projects, the majority of them face many challenges such as: lack of patients’ awareness and acceptance of receiving healthcare services *via* telemedicine applications, shortage of both financial and legalization frameworks, and lack of capacity building programs (El Tokali et al., 2009).

Telemedicine can be useful in providing medical consultations, diagnoses, getting second medical opinion across distances, and providing e-learning for healthcare professionals. It can save patients and physicians travel, time, and money especially in rural areas (Ouma and Herselman, 2008). Fortunately, there are many enabling factors for the provision of telemedicine in Egypt: the presence of an effective ICT infrastructure all over the country, the concept of telemedicine has been proven through MoHP/MCIT cooperation in different eHealth projects, the wide use of mobile technologies, and the availability of both ICT and healthcare capacities (Hussein and Khalifa, 2012). Based on the ITU studies performed in 2008 (International Telecommunication Union ITU, 2008) and WHO studies performed in 2010 (World Health Organization, 2010), the recommendations to facilitate the telemedicine application in Egypt include:

1. developing the needed legislative and administrative frameworks,
2. designing long-term strategic plans to develop eHealth services,
3. adapting a national policy for eHealth,
4. fund research projects in telemedicine with international partners,
5. establish national programs for training healthcare professionals on eHealth solutions

## Future Plans

No real development and progress can be achieved in the healthcare sector without a strong national ICT sector to lead the necessary change (MCIT, 2018). Planning for a new ICT strategy in Egypt is a very promising step towards better healthcare status. New initiatives and development of communications on both national and international levels will be adopted by the new ICT 2030 strategy. Capacity building, designing electronics, and manufacturing are included in the new strategy aiming to maximize the contribution of ICT (MCIT, 2018).

## Conclusion

Health technologies can have a great role in solving the global challenges facing the healthcare systems. Worldwide, healthcare environment is changing due to shortage of healthcare professionals, the growth in chronic illness, and limited resources. Using eHealth applications will help us cope up with that change and will provide patient-focused healthcare and personalized medicine. In Egypt, various eHealth applications have been developed. However, they are still not optimally deployed due to many challenges as financial limitations and lack of ICT infrastructure and national eHealth policy. Therefore, action needs to be started by developing legislative frameworks, adapting a national policy for eHealth and funding research projects. This paper aims to shed the light for the Egyptian policy makers on how new technologies can impact healthcare so that we can start strengthening their quality, releasing new innovations, and paving the way to overcome any challenges.

## Author Contributions

SF fulfills the criteria of authorship and she is the sole author of this paper. She was responsible for the research design, data collection and review, writing and revising the paper, and giving the final approval of the version to be submitted.

## Conflict of Interest Statement

The author declares that the research was conducted in the absence of any commercial or financial relationships that could be construed as a potential conflict of interest.

## Abbreviations

ICT, Information and communication technology; NCDs, Noncommunicable diseases; HER, Health electronic records; ADCs, Automated dispensing cabinets; PACS, National Picture Archiving and Communications System project; RIS, Radiology Information System.
